# Entropy-Based Variational Scheme with Component Splitting for the Efficient Learning of Gamma Mixtures

**DOI:** 10.3390/s22010186

**Published:** 2021-12-28

**Authors:** Sami Bourouis, Yogesh Pawar, Nizar Bouguila

**Affiliations:** 1 Department of Information Technology, College of Computers and Information Technology, Taif University, P.O. Box 11099, Taif 21944, Saudi Arabia; s.bourouis@tu.edu.sa; 2 The Concordia Institute for Information Systems Engineering (CIISE), Concordia University, Montreal, QC H3G 1T7, Canada; yogesh.pawar@mail.concordia.ca

**Keywords:** Gamma mixtures, variational Bayes, entropy, component splitting, texture clustering, objects categorization, gesture recognition

## Abstract

Finite Gamma mixture models have proved to be flexible and can take prior information into account to improve generalization capability, which make them interesting for several machine learning and data mining applications. In this study, an efficient Gamma mixture model-based approach for proportional vector clustering is proposed. In particular, a sophisticated entropy-based variational algorithm is developed to learn the model and optimize its complexity simultaneously. Moreover, a component-splitting principle is investigated, here, to handle the problem of model selection and to prevent over-fitting, which is an added advantage, as it is done within the variational framework. The performance and merits of the proposed framework are evaluated on multiple, real-challenging applications including dynamic textures clustering, objects categorization and human gesture recognition.

## 1. Introduction

The amount of multimedia data available in the world is increasing at an astounding rate. Analyzing these heterogeneous and multimodal data automatically and extracting knowledge instantly through machine learning techniques has become a substantial problem for various decision-making fields. Among the most used techniques are clustering, recognition and classification [1]. For instance, image classification research focuses on seeking effective image representation that can be utilized to categorize images into different categories and then to learn patterns in these classes. Pattern recognition is often applied in the new-age technical sectors, such as human gesture recognition, face identification, speech recognition and so on. Furthermore, clustering techniques aim at grouping items having the same features and this process can assist businesses, for instance, in identifying separate groups within their customer base. These problems have great practical applications in multimedia information retrieval, machine learning, data security and pattern recognition, to name a few. Much research in various decision-making fields and many real-life computer vision applications has been conducted that focuses on finding efficient algorithms to analyze data accurately. Although much research has been carried out, the obtained performance is far from reliable and leaves these issues open, to a large extent, for further investigation. Indeed, how to build an accurate model of high-dimensional data in a compact and reliable way is one of the most difficult issues.

The knowledge of the statistical properties of the data has a crucial role in the majority of applications. Among the main developed methods in this context, finite mixture models have been broadly adopted, thanks to their flexibility [2,3,4,5,6,7,8]. The basic idea is to assume that the data can be represented by a mixture of distributions, of which we then need to estimate the parameters. For instance, the Gaussian mixture model (GMM) has shown its effectiveness in many applications, due to its simplicity in data modeling [9,10,11].

However, when dealing with mixture models, we face the following challenging issues: (i) selecting a flexible distribution that well-describes and fits complex (non-Gaussian) shapes; (ii) accurately estimating the parameters of the probabilistic model; and, finally, (iii) defining the appropriate number of clusters (known also as studying the model complexity). Furthermore, in many cases, complex data cannot be represented by simple Gaussian distributions.

To deal with conventional GMM limitations, many other alternatives have been proposed. Examples include the Gamma (GaM) mixture which has been shown to fit different types of data and to provide better results than GMM  [12,13,14,15] thanks to its long-tailed distributions. In learning statistical mixture model, the most common estimation algorithm is expectation maximization (EM), which is based on the maximum likelihood estimator (MLE) [2,16]. Nevertheless, this estimator suffers from dependency on initialization, may converge to local maxima instead of a global one, and can result in wrong parameters estimation. To overcome such issues, an alternative is using a pure Bayesian approach, such as Markov chain Monte Carlo (MCMC) [17,18], which has proven to be more efficient than MLE, but it is also computationally intensive, and convergence is not always guaranteed. Consequently, to profit from the merits of both pure Bayesian and MLE techniques and avoid their drawbacks, variational Bayes approaches have been proposed as effective alternatives [19,20,21]. In particular, variational approaches are more controllable, are less costly in computation than MCMC, and can efficiently address the problem of overfitting and parameters estimation. The basic idea is to determine the optimal approximation via, for instance, Kullback—Leibler (KL) divergence (i.e., difference between the approximated posterior distribution and the true one) [22].

To accurately determine the number of mixture clusters (this problem is known as model complexity) when dealing with mixture models, some researches have considered different criteria, such as MML and MDL [6]. In other works, the so-called component-splitting criterion has been investigated [9]. The main idea is to begin with two components (clusters) and to then progressively add more components by splitting existing ones. For example, in [23], entropy measures are computed and investigated via variational learning framework to split the components of Gaussian mixture models.

The objective of our work is to investigate the modeling capabilities of Gamma mixtures and to develop a variational approach to learning finite Gamma mixture models. Moreover, we go a step further, by incorporating an entropy metric and component splitting approach to handle the model selection and parameters estimation problems simultaneously. The added advantage of this method is that it happens within a Gamma mixture model and entropy-based variational framework. Thus, it is possible to automatically select the optimal number of clusters to learn the model’s parameters efficiently and to overcome the problem of under-fitting. The merits of the proposed framework are proved through some challenging applications involving dynamic textures clustering, objects categorization and human gesture recognition.

The rest of the paper is organized in the following manner. In Section 2 we introduce the finite Gamma mixture model with local model selection. In Section 3, the details of our variational Bayes learning framework via entropy-based splitting are described. Section 4 is devoted to reporting the obtained results, which are based on several challenging applications, to verify the merits and effectiveness of our framework, and Section 5 concludes the paper.

## 2. The Statistical Model

In this section, a brief description of finite Gamma mixture modeling is presented, then we introduce the mixture model with local model selection using a component-splitting approach. The motivation for choosing Gamma mixtures is mainly due to its flexibility in terms of modeling non-Gaussian and complex shapes, and also its ease of use.

### 2.1. Finite Gamma Mixture Model

Let us suppose we have a data set denoted by Y with *N* data instances $Y=\{{\vec{Y}}_{1},\ldots ,{\vec{Y}}_{N}\}$ (i.e., feature vectors), where each $\vec{Y_{i}}=\left(Y_{i1},Y_{i2},\ldots ,Y_{iD}\right)$ is a *D*-dimensional positive vector that can be modeled using a Gamma distribution:

(1) $$p\left({\vec{Y}}_{i}|\theta \right)=\prod_{d=1}^{D}\frac{\beta_{d}^{\alpha_{d}}\;\;Y_{id}^{\alpha_{d}-1}\;\;e^{-\beta_{d}Y_{id}}}{\Gamma (\alpha_{d})}$$

where ${\vec{Y}}_{i}$ (i=1,…,N) satisfies $0\leq Y_{id}$, for d=1,…,D; $\alpha_{d}$ is the shape and $\beta_{d}$ the location parameter of this distribution (here $\theta =\{\alpha_{d},\beta_{d}\}$). The function Γ(.) is defined as: $\Gamma \left(x\right)=\int_{0}^{\infty }s^{x-1}e^{-s}ds$.

If the *D*-dimensional vector $\vec{Y}$ (observed data) is distributed according to a mixture of Gamma distributions with *M* components, then we have

(2) $$p\left(\vec{Y}|\Theta \right)=\sum_{j=1}^{M}\pi_{j}p\left({\vec{Y}}_{i}|\theta_{j}\right)$$

where the vector $\pi_{j}$ denotes mixing coefficients with the constraints $0⩽\pi_{j}⩽1,$ and $\sum_{j=1}^{M}\pi_{j}=1$. $\Theta =\{\theta_{1},\theta_{2},\ldots ,\theta_{M},\pi_{1},\ldots ,\pi_{M}\}$ and $\theta_{j}=\left\{\alpha_{jd},\beta_{jd}\right\}$ is the set of parameters of the $j^{th}$ mixture component. We now introduce an indicator matrix $Z=({\vec{Z}}_{1},...,{\vec{Z}}_{N})$ which indicates to which component each data sample is assigned. Here $\vec{Z_{i}}=\left(Z_{i1},...,Z_{iM}\right)$. $\vec{Z_{i}}$ is a binary vector that satisfies the conditions $Z_{ij}\in \left\{0,1\right\}$ and $\sum_{j=1}^{M}Z_{ij}=1$, such that $Z_{ij}=1$ if ${\vec{Y}}_{i}\in j$ and $Z_{ij}=0$ otherwise. The conditional distribution of Z can thus be defined as:

(3) $$p\left(Z|\vec{\pi }\right)=\prod_{i=1}^{N}\prod_{j=1}^{M}\pi_{j}^{Z_{ij}}$$

Now, the conditional probability of the data, given Z (class labels), is expressed as

(4) $$p\left(Y|Z,\Theta \right)=\prod_{i=1}^{N}\prod_{j=1}^{M}{\left[p({\vec{Y}}_{i}|\vec{\theta_{j}})\right]}^{Z_{ij}}$$

### 2.2. Finite Gamma Mixture Model with Local Model Selection

In this work, we address the problem of model selection in finite Gamma mixtures using a component-splitting approach, which has been successfully applied for the case of Gaussian and Dirichlet mixtures in [9,24]. Indeed, this approach has the advantage of preventing over-fitting. The core idea of this algorithm is to partition (split) the components on the basis of a split criterion into two different sets: fixed and free components. We constrain the algorithm to perform computations on only the free components and we assume that the fixed components fit the dataset already (fixed components perfectly approximate the data). Let us denote by *s* the free components and let the remaining M−s be the fixed ones. Thus, our framework is developed based on this local model selection design and then we can reformulate the prior distribution of Z in Equation (5) as,

(5) $$p\left(Z|\vec{\pi },\vec{\pi^{*}}\right)=\prod_{i=1}^{N}\left[\prod_{j=1}^{s}\pi_{j}^{Z_{is}}\prod_{j=s+1}^{M}\pi_{j}^{*Z_{ij}}\right]$$

where $\left\{\pi_{j}\right\}$ and $\left\{\pi_{j}^{*}\right\}$ indicate the mixing coefficients of the free and fixed components, respectively. It is to noted that $\left\{\pi_{j}\right\}$, $\left\{\pi_{j}^{*}\right\}$ > 0 and follow the constraint:

(6) $$\sum_{j=1}^{s}\pi_{j}+\sum_{j=s+1}^{M}\pi_{j}^{*}=1$$

Subsequently, we need to introduce a prior over $\left\{\pi_{j}^{*}\right\}$ (fixed mixing coefficient). It is noted that $\left\{\pi_{j}^{*}\right\}$ are considered random variables. The goal here is to find the conditional probability of fixed components that depends only on the free mixing coefficients $\left\{\pi_{j}\right\}$. As introduced in [9], we choose a prior for $\vec{\pi_{j}^{*}}$ as a non-standard Dirichlet distribution.

(7) $$p\left(\vec{\pi^{*}}|\vec{\pi }\right)={\left(1-\sum_{k=1}^{s}\pi_{k}\right)}^{-M+s}\frac{\Gamma (\sum_{j=s+1}^{M}c_{j})}{\prod_{j=s+1}^{M}\Gamma \left(c_{j}\right)}\prod_{j=s+1}^{M}{\left(\frac{\pi_{j}^{*}}{1-\sum_{k=1}^{s}\pi_{k}}\right)}^{c_{j}-1}$$

Next, conjugate priors have to be determined for the model’s parameters. Unfortunately, in our case, there are no possible priors. Thus, based on the fact that our parameters are positive and statistically independent, the Gamma distribution is an appropriate choice to approximate these priors $(\vec{\alpha }$ and $\vec{\beta })$. They can be expressed as:

(8) $$p\left(\vec{\alpha }\right)=G\left(\vec{\alpha }|\vec{u},\vec{v}\right)=\prod_{j=1}^{M}\prod_{l=1}^{D}G\left(\alpha_{jl}|u_{jl},v_{jd}\right)=\prod_{j=1}^{M}\prod_{l=1}^{D}\frac{v_{jl}^{u_{jl}}}{\Gamma (u_{jl})}\alpha_{jl}^{u_{jl}-1}e^{-v_{jl}\alpha_{jl}}$$

(9) $$p\left(\vec{\beta }\right)=G\left(\vec{\beta }|\vec{g},\vec{h}\right)=\prod_{j=1}^{M}\prod_{l=1}^{D}G\left(\beta_{jd}|g_{jl},h_{jl}\right)=\prod_{j=1}^{M}\prod_{l=1}^{D}\frac{h_{jl}^{g_{jl}}}{\Gamma (g_{jl})}\beta_{jl}^{g_{jl}-1}e^{-h_{jl}\beta_{jl}}$$

Finally, the joint distribution of all the random variables is determined as follows:

(10) $$\begin{array}{cc} p(Y,Z,\vec{\alpha },\vec{\beta },\vec{\pi^{*}}|\vec{\pi }) & =p\left(Y|Z,\vec{\alpha },\vec{\beta }\right)p\left(Z|\vec{\pi },\vec{\pi^{*}}\right)p\left(\vec{\pi^{*}}|\vec{\pi }\right)p\left(\vec{\alpha }\right)p\left(\vec{\beta }\right)\\ \; & =\prod_{i=1}^{N}\prod_{j=1}^{M}{\left[\prod_{d=1}^{D}\frac{\beta_{d}^{\alpha_{d}}\;\;Y_{id}^{\alpha_{d}-1}\;\;e^{-\beta_{d}Y_{id}}}{\Gamma (\alpha_{d})}\right]}^{z_{ij}}\\ \; & \times \prod_{i=1}^{N}\left[\prod_{j=1}^{s}\pi_{j}^{Z_{is}}\prod_{j=s+1}^{M}\pi_{j}^{*Z_{ij}}\right]\\ \; & \times {\left(1-\sum_{k=1}^{s}\pi_{k}\right)}^{-M+s}\frac{\Gamma (\sum_{j=s+1}^{M}c_{j})}{\prod_{j=s+1}^{M}\Gamma \left(c_{j}\right)}\prod_{j=s+1}^{M}{\left(\frac{\pi_{j}^{*}}{1-\sum_{k=1}^{s}\pi_{k}}\right)}^{c_{j}-1}\\ \; & \times \prod_{j=1}^{M}\prod_{l=1}^{D}\frac{v_{jl}^{u_{jl}}}{\Gamma (u_{jl})}\alpha_{jl}^{u_{jl}-1}e^{-v_{jl}\alpha_{jl}}\\ \; & \times \prod_{j=1}^{M}\prod_{l=1}^{D}\frac{h_{jl}^{g_{jl}}}{\Gamma (g_{jl})}\beta_{jl}^{g_{jl}-1}e^{-h_{jl}\beta_{jl}} \end{array}$$

It is noteworthy that free coefficients are considered, here, parameters and not random variables; therefore, we do not place a prior over $\vec{\pi }$.

## 3. Variational Bayesian Learning via Entropy-Based Splitting

### 3.1. Model Learning Using Variational Bayes

For the parameter estimation problem, we focus, here, on the application of variational Bayes with the mean field approximation, which has been shown to be an efficient technique for inferring posterior distributions of mixture models [20,25,26]. Indeed, variational Bayes has been proposed as an efficient solution for posteriors approximation with low computational cost, as opposed to other inference approaches such as the MCMC technique [8,27]. Due to the computational complexity of the true posterior p(Θ∣Y), the best methodology to follow is to find a good approximation for it, which we denote by Q(Θ), that can be calculated easily [20]. Indeed, p(Θ∣Y) is known to be intractable and cannot be calculated directly. Accordingly, we propose determining this approximation by maximizing the lower bound, ln(p(Y)), as follows:   

(11) $$L\left(Q\right)=\int Q\left(\Theta \right)\;ln\left(\frac{p(Y,\Theta )}{Q(\Theta )}\right)d\Theta$$

where $\Theta =\{Z,\vec{\pi },\vec{\alpha },\vec{\beta }\}$ includes both latent variables and random parameters. Next, we factorize the distribution Q(Θ) into disjoint tractable distributions by using the mean field theory, as in [1]. This process leads to the following expression:

(12) $$Q\left(\Theta \right)=Q\left(Z,\vec{\pi },\vec{\alpha },\vec{\beta }\right)=Q\left(Z\right)Q\left(\vec{\pi }\right)Q\left(\vec{\alpha }\right)Q\left(\vec{\beta }\right)$$

Finally, the solutions of the updated variational posteriors are obtained by optimizing L(Q) with respect to each distribution. The resulting solutions are expressed as follows:

(13) $$Q\left(Z\right)=\prod_{i=1}^{N}\prod_{j=1}^{M}r_{ij}^{Z_{ij}}$$

(14) $$Q\left(\vec{\pi }\right)=Dir\left(\vec{\pi }|a_{0}\right)$$

(15) $$Q\left(\vec{\alpha }\right)=\prod_{j=1}^{M}\prod_{d=1}^{D}G\left(\alpha_{jd}|u_{jd}^{*},v_{jd}^{*}\right)$$

(16) $$Q\left(\vec{\beta }\right)=\prod_{j=1}^{M}\prod_{d=1}^{D}G\left(\alpha_{jd}|g_{jd}^{*},h_{jd}^{*}\right)$$

where the hyperparameters in the above equations can be fixed in a similar way as in [26] by testing and experimenting different values depending on the data set to model .

### 3.2. Gamma Model Learning via Entropy-Based Component Splitting

In this section, we develop a robust variational learning approach through the entropy-based splitting method to learn the Gamma mixture model. We are fundamentally encouraged by the entropy principle, as suggested in [23], to learn Gaussian mixtures. The core idea is to evaluate the quality of fitting of a component of the implemented Gamma mixture model. Thus, it is possible to evaluate the goodness of fitting components of such a model. This step is achieved by making a comparison between the theoretical entropy and the estimated entropy. In particular, we proceed by calculating an estimation of the entropy using MeanNN entropy [28] and then compare it with the theoretical maximum entropy to check if a component is truly distributed with Gamma. In case of a significant difference (greater than $10^{-2}$), we can conclude that this component does not fit well and so we proceed with a portioning process which leads to the division of the current component into two new clusters. As a result, via the proposed entropy-based learning approach for Gamma mixtures, we can assess accurately the number of components (i.e., define model complexity) by making a comparison between the estimated and theoretical entropies.

#### 3.2.1. Theoretical Entropy of Gamma Mixtures

Let us denote, by $\vec{Y_{i}}$, a continuous random variable and, by $p(\vec{Y})$, its probability density function; then the expression of the differential entropy of $\vec{Y}$ is given, as in [29], by:   

(17) $$H\left(\vec{Y_{i}}\right)=-\int p\left(\vec{Y_{i}}\right)\;ln\;p\left(\vec{Y_{i}}\right)d\vec{Y_{i}}$$

In our case, $\vec{Y}$ is supposed distributed according to a Gamma distribution (given in Equation (1)). After simplification, we obtain the following theory value of the maximum differential entropy of $\vec{Y}$, given as:

(18) $$H_{Ga}\left(\vec{Y}\right)=-\sum_{d=1}^{D}ln\;\left(\beta_{d}\right)+\sum_{d=1}^{D}ln\;\left(\Gamma \left(\alpha_{d}\right)\right)-\sum_{d=1}^{D}\left(\alpha_{d}-1\right)\psi \left(\alpha_{d}\right)+\sum_{d=1}^{D}\alpha_{d}$$

where ψ is a digamma function, such as $\psi \left(x\right)=\frac{d}{dx}ln\;\left(\Gamma \left(x\right)\right)$

#### 3.2.2. MeanNN Entropy Estimator

In order to assess if a given component is truly distributed according to a Gamma distribution, we proceed with an estimator, namely, MeanNN entropy, proposed in [28]. It is an extension to the Shannon entropy that allows estimating the entropy $H(\vec{Y})$ of a D-dimensional random variable $\vec{Y_{i}}$ by supposing we have an unknown density function $p(\vec{Y_{i}})$ [30]. The Shannon differential entropy, given in Equation (17), is applied. By estimating $ln\;p(\vec{Y_{i}})$, we can determine an unbiased entropy estimator. We follow the key idea in [28], where ϵ is the diameter of a ball centered at $\vec{Y_{i}}$. We suppose that there exists a point within the distance of [ϵ,ϵ+dϵ]. Therefore, it is possible to discover other points having smaller $\left(\hat{k}-1\right)$ or larger $\left(N-\hat{k}-1\right)$ distances from $\vec{Y_{i}}$. Based on this paradigm, the distance probability function to be satisfied (i.e., between $\vec{Y_{i}}$ and its ${\hat{k}}^{th}$ nearest neighbor) is given as:

(19) $$p_{i\hat{k}}\left(\epsilon \right)=\frac{(N-1)!}{\left(\hat{k}-1\right)!\left(N-\hat{k}-1\right)!)}\frac{dp_{i}\left(\epsilon \right)}{d\epsilon }p_{i}^{\hat{k}-1}{\left(1-p_{i}\right)}^{\left(N-\hat{k}-1\right)}$$

$p_{i}\left(\epsilon \right)$ represents the ϵ-ball mass centered at $\vec{Y_{i}}$:

(20) $$p_{i}\left(\epsilon \right)=\int_{\left|\right|\vec{Y}-\vec{Y_{i}}\left|\right|}p\left(\vec{Y_{i}}\right)d\vec{Y_{i}}$$

the expected value of $logp_{i}\left(\epsilon \right)$ with respect to $p_{i}\left(\epsilon \right)$ is given:

(21) $$E\left(logp_{i}\left(\epsilon \right)\right)=\int_{0}^{\infty }p_{i\hat{k}}logp_{i}\left(\epsilon \right)d\epsilon =\psi \left(\hat{k}\right)-\psi \left(N\right)$$

In the whole ϵ-ball, p($\vec{Y_{i}}$) is supposed to be constant. So, we have:

(22) $$p_{i}\left(\epsilon \right)≃V_{d}\epsilon^{d}p\left(\vec{Y_{i}}\right)$$

(23) $$V_{d}=\frac{\pi^{d/2}}{\Gamma (1+d/2)}$$

where *d* is the dimension of $\vec{Y_{i}}$ and $V_{d}$ denotes the unit ball volume. When substituting Equation (22) into Equation (21), $-logp(\vec{Y_{i}})$ is determined as

(24) $$-logp\left(\vec{Y_{i}}\right)≃\psi \left(N\right)-\psi \left(\hat{k}\right)+dE\left(log\;\epsilon \right)+logV_{d}$$

which leads to the unbiased kNN estimator of the differential entropy as

(25) $$H_{\hat{k}}\left(\vec{Y}\right)=\psi \left(N\right)-\psi \left(\hat{k}\right)+\frac{d}{N}\sum_{i=1}^{N}log\;\epsilon_{i}+logV_{d}$$

Based on the assumption in [23], the differential entropy can be extracted from the mean of many estimators corresponding to different values of k. Thus, if we consider all values of k (i.e., from 1 to N−1) , we obtain the following result of the differential entropy:

(26) $$H_{M}\left(\vec{Y}\right)=\frac{1}{N-1}\sum_{\hat{k}=1}^{N-1}H_{\hat{k}}\left(\vec{Y}\right)=logV_{d}+\psi \left(N\right)+\frac{1}{N-1}\sum_{\hat{k}=1}^{N-1}\left[\frac{d}{N}\sum_{i=1}^{N}log\;\epsilon_{i,\hat{k}}-\psi \left(\hat{k}\right)\right]$$

where $\epsilon_{i,\hat{k}}$ is the $\hat{k}$-th nearest neighbor of $\vec{Y_{i}}$.

The maximum entropy of the our Gamma mixture model can be expressed by

(27) $$H_{Ga}\left(\vec{Y}\right)=\sum_{j=1}^{M}\pi_{j}H_{Ga}\left(j\right)$$

where $H_{Ga}\left(j\right)$ is the maximum differential entropy of the $j^{th}$ cluster.

Thereafter, we can assess the quality of fitting the developed model within each cluster while comparing the entropy mentioned above. Indeed, we denote, by $\Omega_{Ga}$, the results of calculating the normalized, weighted sum of the difference between two entropies as in [23]. This output is evaluated for each component of our model, GaMM, as:

(28) $$\Omega_{Ga}=\sum_{j=1}^{M}\pi_{j}\left[\frac{H_{Ga}\left(j\right)-H_{M}\left(j\right)}{H_{Ga}\left(j\right)}\right]=\sum_{j=1}^{M}\pi_{j}\left[1-\frac{H_{M}\left(j\right)}{H_{Ga}\left(j\right)}\right]$$

where $H_{M}\left(j\right)$ is the result entropy for the component *j*, which is computed by the MeanNN estimator. Thus, $\Omega_{Ga}$ is in the interval [0,1]. If the observed dataset is truly Gamma distributed, then the value of $\Omega_{Ga}$ reaches to zero. Now the splitting process is based on selecting the component $j^{*}$ with the highest $\Omega_{Ga}\left(j\right)$ as follow:

(29) $$j^{*}=\arg\max_{j}\left[\Omega_{Ga}\left(j\right)\right]=\arg\max_{j}\left[\pi_{j}\frac{H_{Ga}\left(j\right)-H_{M}\left(j\right)}{H_{Ga}\left(j\right)}\right]$$

Thus, we inspect $\Omega_{Ga}$ by comparing both the theoretical and estimated entropies of the Gamma mixture, then we split $j^{*}$ into two new components.

#### 3.2.3. Variational Learning Algorithm via Entropy-Based Splitting

The proposed variational inference algorithm for Gamma mixture models is illustrated in Algorithm 1. It is noteworthy that there are two scenarios for our algorithm. In the first one, all components are kept (i.e., all mixing coefficients are different from zero). In this case, the splitting process will be performed with success and the number of clusters (components) will be increased by one (K+1). The component that will be selected to be split into two new clusters is the one that has the largest $\Omega_{Ga}\left(j\right)$. The second scenario happens when one of the mixing coefficients is near zero. In this case, its associated component will be deleted (K−1), the splitting process is not performed, and the algorithm is stopped with *k* clusters. Note that we start with one component (M=1).
**Algorithm 1:**Proposed Entropy-based Variational Learning for GaMM.(1) **Initialization**          Initialize hyperparameters *u*, *v*, *g*, *h*, *a*${}_{0}$.(2) **Splitting process**     Split *j*${}^{*}$ into two new components *j*${}_{1}$ and *j*${}_{2}$ with equal proportion equal $\pi^{*}$/2    •M = M + l    • Initialise the parameters of *j*${}_{1}$ and *j*${}_{2}$ using same parameters of *j*${}^{*}$(3) Perform standard variational Bayes, until convergence.(4) Determine the number of components through the evaluation of the mixing coefficients {$\pi_{j}$}(5) **if** *$\pi_{j}⋍0$***then**((*M = M*−*1* and program terminates
**end**(**else**((Evaluate $\Omega_{Ga}$, choose *j*${}^{*}$ according to Equation (29), and go to the splitting process in step**end**(

## 4. Experimental Results

### 4.1. Dynamic Texture Clustering

Dynamic textures (DT) are defined by Doretto et al. [31] as an extension of texture to the temporal domain. In other words, it is a sequence of images of moving scenes that display specific stationary properties in time (e.g., smoke, clouds, sea waves and trees). In such case, the spatial (i.e., appearance) and temporal (i.e., motion) characteristics may not be the same. DT plays a substantial role in many applications and the modeling of DT has been addressed by many researchers to solve different problems, including motion synthesis or retrieval, motion classification, recognition and segmentation [32]. Thus, new concepts that can be derived from static texture approaches are needed to integrate the analysis of temporal variations into the spatial analysis. However, the main issues encountered in dynamic textures analysis arise from the large range of appearances and the association of both temporal and spatial properties.

In order to apply the proposed entropy-based learning model to clustering dynamic textures, some preprocessing steps are performed. First, we start by extracting spatial visual features with scale-invariant feature transform (SIFT) [33], which is largely utilized in such contexts. In order to encode the full dynamic texture, including time information, SIFT is considered insufficient. Furthermore, we propose taking into account other temporal descriptors, such as the so-called space-time interest points (STIPs) [34]. As a result, we calculate 128-dimensional SIFT/STIPs descriptors from each frame of every video through the difference-of-Gaussians (DoG) detector. This step allows the selection of potential interest points in which we ensure both rotation and scale invariance. Next, these features are combined with features extracted using the imagenet trained deep learning model and, finally, normalized and modeled using the developed (GaM-En) approach. It is noted that we do not need to consider the class labels because our aim is to perform clustering analysis in an unsupervised manner.

**Figure 1:**
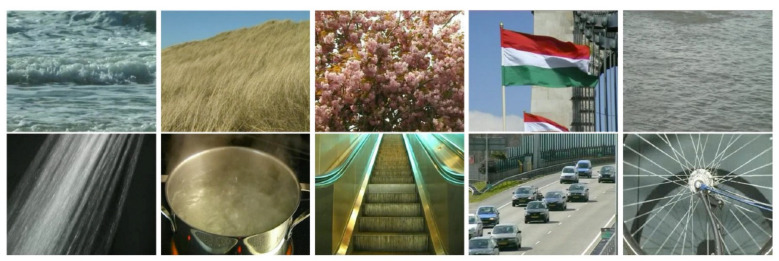
Sample snapshots from different categories of the DynTex dataset.

In this experiment, a challenging dynamic texture dataset, the DynTex database [35], is used to evaluate performance. It consists of more than 650 dynamic texture sequences videos, in PAL format (720 × 576, 25 fps). In this work, we limit our work to a subset of videos representing 10 different categories, including flags, sea, vegetation, clam water, trees, smoke, fountains, fountains, traffic, fountains and rotation. Every category contains 20 videos. Some samples from these categories are depicted in Figure 1.

In order to assess the performance of GaM-EN, we compare it with three other methods; the Gaussian mixture model with a component-splitting technique (GM-Split), the Gaussian mixture model via entropy-based learning (GM-EN) and the Gamma mixture model via variational-based learning (GaM-VB). Thus, we have run each testing method 30 times and report the average results in terms of accuracy and processing time. The averages of the clustering accuracy are given in Table 1. From these results, GaM-En has reached 93.40%; however, the accuracies of the others are less than 88%, which confirms that our model is able to provide better performance. This fact demonstrates a significant improvement when using Gamma distribution and entropy-based variational learning over Gaussian-based models to distinguish dynamic texture categories.

### 4.2. Human Gesture Recognition

Recognizing human gestures has become an important active research direction in the fields of computer vision and pattern recognition that may be applied in many potential applications, such as human–computer interaction, artificial intelligence, video surveillance systems, virtual reality, etc. Indeed, human gestures (or actions) are the natural way of expressing intentions in people’s daily lives. The use of gestures can help people with certain disabilities to communicate with others. In particular, hand recognition is a technique that helps in understanding the movement of a hand. Recently, this research field has been gaining increasing attention and, so far, many research works have been conducted on human gesture recognition [16,36,37,38]. Nevertheless, it still remains a challenging research field, primarily due to the complexity and ambiguity of human motion and of backgrounds. The goal of this experiment is to evaluate our proposed statistical approach (GaM-En) with two types of human gesture recognition, which are hand and body gestures. In this experiment, we proceed as in [39] in order to obtain discriminative features for gesture detection in the spatiotemporal domain from each video. Indeed, both motion and appearance features are extracted for human gestures characterization. For motion features, we use the so-called motion history image (MHI) [40]. Then, the histogram of oriented gradients features (HOG) [41] is adopted for extracting appearance feature, which takes into account the magnitude of edge, direction and corner information. Finally, we apply the model of bag-of-visual words to quantize the resulting discriminative vectors via the K-means algorithm. As a result, a histogram vector (representing the frequency of each visual word) is constructed to model each input frame. After this preprocessing step, we apply our proposed statistical model (GaM-En) to recognize human gestures. In particular, each test video is assigned to the appropriate category with the maximum posterior probability under Bayes’ rule.

**Figure 2:**
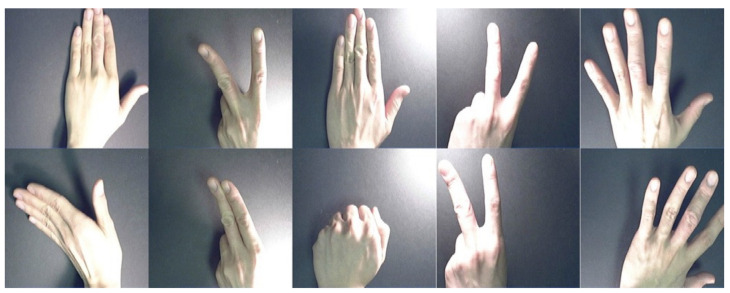
Sample frames of hand gesture from Cambridge-Gesture dataset.

In this experiment (hand gesture recognition), we consider the Cambridge-Gesture database [42] as a public database. It includes nine hundred (900) image sequences representing nine different classes of hand gesture data. These classes are composed of three primitive hand shapes (‘Flat’, ‘Spread’ and ‘Vshape’) and three primitive motions (leftward, rightward and contracting). In every class there are 100 sequences captured with different illuminations and arbitrary motions, and the size of each image is 320 × 240 pixels. In our case, the dataset is divided into two equal partitions: one is used for training and the other is for testing. Sample hand gesture frames from this database can be viewed in Figure 2.

**Figure 3:**
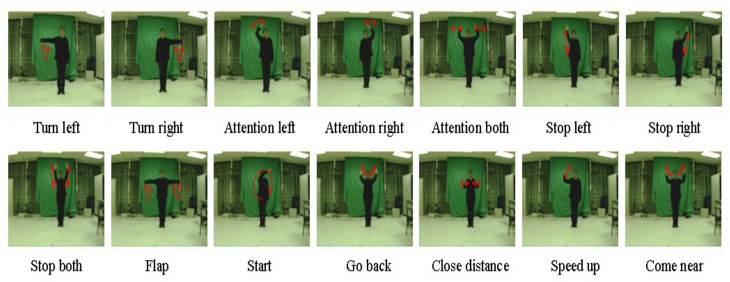
Sample frames of body gestures (gesture classes) from the UMD Keck body-gesture dataset.

We also conduct other experiments on human body gesture recognition and we test our approach using the publicly available dataset UMD Keck body-gesture (http://www.umiacs.umd.edu/~zhuolin/Keckgesturedataset.html, accessed on 5 December 2021) [43]. This database includes 14 different gesture classes: Turn Left (A), Turn Right (B), Attention Left (C), Attention Right (D), Attention Both (E), Stop Left (F), Stop Right (G), Stop Both (H), Flap (I), Start (J), Go Back (K), Close Dist (L), Speed Up (M), Come Near (N). It comprises people representing a subset of military signals. Thus, we have 126 video sequences captured by fixed camera and 168 videos collected from dynamic environments. Sample body gesture frames from the UMD Keck dataset are shown in Figure 3.

In order to demonstrate the benefits of using Gamma models with entropy-based variational learning and component splitting for both body and hand gesture recognition, we calculate the confusion matrix for the UMD Keck body-gesture database. Furthermore, we compare the performance on the Cambridge-Gesture dataset through the overall recognition accuracy. This is performed for our approach (GaM-En) and three other mixture methods, Gaussian mixture model with component splitting technique (GM-Split), Gaussian mixture model via entropy-based learning (GM-En), and Gamma mixture model via variational-based learning (GaM-VB). Table 2 reports the average results obtained by testing different approaches 30 times for accuracy and processing time. Based on this comparative study, we can see clearly that our model has a higher overall recognition accuracy (91.66%) than the others. Moreover, the shortest required processing time to reach the optimal solution is obtained with the proposed GaM-En. For the other models, the accuracy is less than 87%. These results prove again the effectiveness of using our entropy-based framework for recognizing human gestures.

### 4.3. Object Categorization

Our last experiment involves the application of object categorization. Indeed, the detection of real-world objects has been an important application of computer vision due to the increasingly huge amounts of images created every day [44,45]. The goal of object categorization is to differentiate the classes of objects from each other. This problem is considered to be difficult due to the changes in viewpoint and illumination conditions that can drastically modify a particular object’s appearance. Several research works have tackled the problem of modeling and categorization objects because solving it will help further tasks in pattern recognition and computer vision applications, such as image classification and retrieval. We address, here, this challenging problem and evaluate the performance of our framework by comparing it with other methods. In particular, our aim is to test the effectiveness of our statistical model in terms of clustering the input from a set of images.

**Figure 4:**
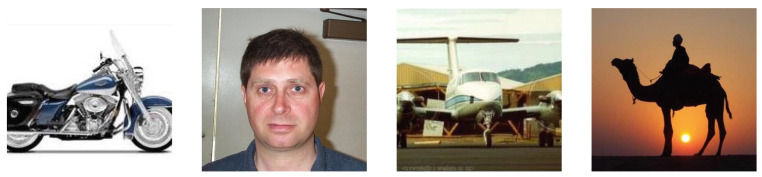
Sample from the Caltech dataset. From left to right: Bikes, Faces, Planes, Camels.

**Figure 5:**
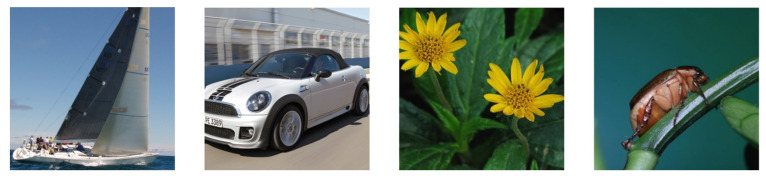
Sample from the GHIM10K dataset. From left to right: Boats, Cars, Flowers, Bugs.

In this section, we evaluate our framework on the basis of two challenging databases, Caltech256 [46] and GHIM10K (http://www.ci.gxnu.edu.cn/cbir/dataset.aspx, accessed on 1 August 2021). Caltech256 contains 600 images divided into four categories: Faces, Planes, Bikes and Camels. The GHIM10K dataset contains 400 images divided into four classes, which are Flowers, Boats, Cars and Bugs. Each class consists of 100 images. To make the problem more challenging, the objects are acquired with different lighting, from different angles and against different background conditions. Samples from these two databases are presented in Figure 4 and Figure 5.

Generally, when addressing the problem of object categorization the first step is to extract robust descriptors from input data. Thus, a preprocessing step was adopted here to extract visual features using SIFT (scale-invariant feature transform). All extracted local SIFT descriptors are grouped into a collection (corpus). Then, K-means is applied to cluster the corpus and generate a visual words vocabulary. In this experiment, the optimal number of vocabulary words is 50.

In order to prove the merits of the proposed framework for object categorization application, we also evaluate other generative model-based methods, such as Gaussian mixture model with component splitting technique (GM-Split), Gaussian mixture model via entropy-based learning (GM-EN), and Gamma mixture model via variational-based learning (GaM-VB). Furthermore, we compare the performance and report the average results from 30 runs in terms of overall categorization accuracy in Table 3. To initialize the model’s parameters, different parameter setting are considered to ensure the robustness of our choice. As illustrated in Table 3, we may notice the merits of GaM-En in differentiating different objects from the Caltech256 and GHIM10K datasets by obtaining the highest accuracy rates: 97.84% and 97.02%, respectively. Lower rates of categorization accuracy are obtained by Gaussian-based models (GM-En and GM-Split). These results demonstrate that entropy-based Gamma offers better modeling capabilities over Gaussian-based models when dealing with compositional feature vectors. On the other hand, it is clear from the same depicted table that entropy-based variational learning (GaM-En) outperforms conventional variation (GaM-VB) in learning Gamma mixture models.

## 5. Conclusions

This paper has presented a novel entropy-based variational approach with a splitting method to learn the parameters of Gamma mixture models. The main goal is to investigate entropy criteria in order to evaluate whether a given component is truly Gamma distributed. This process is performed by comparing theoretical maximum entropy with that calculated by the MeanNN estimator. Subsequently, in the case of having important comparison difference (i.e., we inspect the component with the highest difference), a splitting process is performed and such component is split into two new components (or clusters), since it is not well-fitted by the mixture model. Our developed framework (GaM-En) leads to a principled solution and has the advantage of avoiding over- and under-fitting issues. Through extensive experimentation, including examining the problems of dynamic texture clustering, human gesture recognition and object categorization, we have validated our framework. The obtained results show that our approach is competitive and outperforms some state-of-the-art methods, thanks to its flexibility and effectiveness in terms of multidimensional data modelling and learning. The developed approach has attractive simplicity and generality that makes it easily applied to many other challenging problems, including text clustering and medical image analysis. To improve the expected results, a promising future work could be the integration of a visual feature selection mechanism into the current framework. We plan also to deal with dynamic data by suggesting an online learning process, instead of batch learning. 

## Figures and Tables

**Table 1:** Overall accuracy (%± standard error) of dynamic texture clustering of different approaches using SIFT features on the DynTex dataset.

Approach	Average Accuracy (%) ± Standard Error	Average Time (S)
GM-Split	86.11 ± 1.21	4.26
GM-En	86.34 ± 1.13	4.22
GaM-VB	88.27 ± 1.09	3.56
**GaM-En (our method)**	93.40 ± 1.03	1.64

**Table 2:** The average recognition rate (%±standard error) of hand gestures, using different approaches, performed on Cambridge-Gesture dataset.

Approach	Average Accuracy (%)	Average Time (S)
GM-Split	85.25 ± 1.33	2.21
GM-En	85.28 ± 1.24	2.33
GaM-VB	87.37 ± 1.09	3.18
**GaM-En (our method)**	91.66 ± 0.91	1.59

**Table 3:** Results of object categorization using different models (average %± standard error (Average time (S))).

Datasets/Method	GaM-En (Proposed Method)	GaM-VB	GM-En	GM-Split
Caltech256	97.84 ± 0.86 (2.11)	94.32 ± 1.14 (2.99)	92.97 ± 1.09 (2.28)	92.91 ± 1.10 (2.18)
GHIM10K	97.02 ± 0.92 (2.89)	95.37 ± 1.18 (3.11)	93.33 ± 1.13 (2.97)	93.17 ± 1.15 (2.93)

## Data Availability

Not applicable.

## References

[1] Bishop C.M. (2006). Pattern Recognition and Machine Learning.

[2] Bouguila N., Fan W. (2020). Mixture Models and Applications.

[3] McNicholas P.D. (2016). Model-Based Clustering. J. Classif..

[4] Andrews J.L., McNicholas P.D., Subedi S. (2011). Model-based classification via mixtures of multivariate t-distributions. Comput. Stat. Data Anal..

[5] Liu X., Fu H., Jia Y. (2008). Gaussian mixture modeling and learning of neighboring characters for multilingual text extraction in images. Pattern Recognit..

[6] Bourouis S., Channoufi I., Alroobaea R., Rubaiee S., Andejany M., Bouguila N. (2021). Color object segmentation and tracking using flexible statistical model and level-set. Multim. Tools Appl..

[7] Alharithi F.S., Almulihi A.H., Bourouis S., Alroobaea R., Bouguila N. (2021). Discriminative Learning Approach Based on Flexible Mixture Model for Medical Data Categorization and Recognition. Sensors.

[8] Bourouis S., Alroobaea R., Rubaiee S., Andejany M., Almansour F.M., Bouguila N. (2021). Markov Chain Monte Carlo-Based Bayesian Inference for Learning Finite and Infinite Inverted Beta-Liouville Mixture Models. IEEE Access.

[9] Constantinopoulos C., Likas A. (2007). Unsupervised Learning of Gaussian Mixtures Based on Variational Component Splitting. IEEE Trans. Neural Netw..

[10] Najar F., Bourouis S., Bouguila N., Belghith S. (2020). A new hybrid discriminative/generative model using the full-covariance multivariate generalized Gaussian mixture models. Soft Comput..

[11] Song Z., Ali S., Bouguila N., Fan W. (2020). Nonparametric hierarchical mixture models based on asymmetric Gaussian distribution. Digit. Signal Process..

[12] Beckmann C., Woolrich M., Smith S. (2003). Gaussian/Gamma mixture modelling of ICA/GLM spatial maps. Neuroimage.

[13] Sallay H., Bourouis S., Bouguila N. (2021). Online Learning of Finite and Infinite Gamma Mixture Models for COVID-19 Detection in Medical Images. Computers.

[14] Lai Y., Cao H., Luo L., Zhang Y., Bi F., Gui X., Ping Y. (2021). Extended variational inference for gamma mixture model in positive vectors modeling. Neurocomputing.

[15] Bourouis S., Sallay H., Bouguila N. (2021). A Competitive Generalized Gamma Mixture Model for Medical Image Diagnosis. IEEE Access.

[16] Najar F., Bourouis S., Zaguia A., Bouguila N., Belghith S. Unsupervised Human Action Categorization Using a Riemannian Averaged Fixed-Point Learning of Multivariate GGMM. Proceedings of the Image Analysis and Recognition—15th International Conference, ICIAR 2018.

[17] Evans M., Swartz T. (1995). Methods for approximating integrals in statistics with special emphasis on Bayesian integration problems. Stat. Sci..

[18] Rasmussen C.E., Touretzky D.S., Mozer M., Hasselmo M.E. (1995). A Practical Monte Carlo Implementation of Bayesian Learning. Advances in Neural Information, Proceedings of the Systems 8, NIPS, Denver, CO, USA, 27–30 November 1995.

[19] Corduneanu A., Bishop C.M. (2001). Variational Bayesian model selection for mixture distributions. Artificial Intelligence and Statistics.

[20] Jordan M.I., Ghahramani Z., Jaakkola T.S., Saul L.K. (1999). An Introduction to Variational Methods for Graphical Models. Mach. Learn..

[21] Fan W., Sallay H., Bouguila N., Bourouis S. (2016). Variational learning of hierarchical infinite generalized Dirichlet mixture models and applications. Soft Comput..

[22] Bernardo J., Bayarri M., Berger J., Dawid A., Heckerman D., Smith A., West M. (2003). The variational Bayesian EM algorithm for incomplete data: With application to scoring graphical model structures. Bayesian Stat..

[23] Benavent A.P., Escolano F. (2012). Entropy-Based Incremental Variational Bayes Learning of Gaussian Mixtures. IEEE Trans. Neural Netw. Learn. Syst..

[24] Fan W., Bouguila N., Ziou D. (2014). Variational learning of finite Dirichlet mixture models using component splitting. Neurocomputing.

[25] Fan W., Bouguila N. (2013). Variational learning of a Dirichlet process of generalized Dirichlet distributions for simultaneous clustering and feature selection. Pattern Recognit..

[26] Fan W., Bouguila N., Bourouis S., Laalaoui Y. (2018). Entropy-based variational Bayes learning framework for data clustering. IET Image Process..

[27] Marin J.M., Robert C. (2007). Bayesian Core: A Practical Approach to Computational Bayesian Statistics.

[28] Faivishevsky L., Goldberger J. ICA based on a Smooth Estimation of the Differential Entropy. Proceedings of the Twenty-Second Annual Conference on Neural Information Processing Systems.

[29] Fan W., Bouguila N., Ziou D. (2013). Unsupervised Hybrid Feature Extraction Selection for High-Dimensional Non-Gaussian Data Clustering with Variational Inference. IEEE Trans. Knowl. Data Eng..

[30] Leonenko N., Pronzato L., Savani V. (2008). A class of Rényi information estimators for multidimensional densities. Ann. Stat..

[31] Doretto G., Chiuso A., Wu Y.N., Soatto S. (2003). Dynamic textures. Int. J. Comput. Vis..

[32] Ravichandran A., Chaudhry R., Vidal R. (2013). Categorizing Dynamic Textures Using a Bag of Dynamical Systems. IEEE Trans. Pattern Anal. Mach. Intell..

[33] Lowe D.G. (2004). Distinctive Image Features from Scale-Invariant Keypoints. Int. J. Comput. Vis..

[34] Laptev I., Lindeberg T. Space-time Interest Points. Proceedings of the 9th IEEE International Conference on Computer Vision (ICCV 2003).

[35] Péteri R., Fazekas S., Huiskes M.J. (2010). DynTex: A comprehensive database of dynamic textures. Pattern Recognit. Lett..

[36] Lui Y.M. (2012). Human gesture recognition on product manifolds. J. Mach. Learn. Res..

[37] Mitra S., Acharya T. (2007). Gesture Recognition: A Survey. IEEE Trans. Syst. Man Cybern. Part C.

[38] Fan W., Sallay H., Bouguila N., Bourouis S. (2015). A hierarchical Dirichlet process mixture of generalized Dirichlet distributions for feature selection. Comput. Electr. Eng..

[39] Hu Y., Cao L., Lv F., Yan S., Gong Y., Huang T.S. Action detection in complex scenes with spatial and temporal ambiguities. Proceedings of the 2009 IEEE 12th International Conference on Computer Vision.

[40] Davis J.W., Bobick A.F. The Representation and Recognition of Human Movement Using Temporal Templates. Proceedings of the 1997 Conference on Computer Vision and Pattern Recognition (CVPR ’97).

[41] Dalal N., Triggs B. Histograms of Oriented Gradients for Human Detection. Proceedings of the 2005 IEEE Computer Society Conference on Computer Vision and Pattern Recognition (CVPR 2005).

[42] Kim T., Wong S., Cipolla R. Tensor Canonical Correlation Analysis for Action Classification. Proceedings of the 2007 IEEE Computer Society Conference on Computer Vision and Pattern Recognition (CVPR 2007).

[43] Lin Z., Jiang Z., Davis L.S. Recognizing actions by shape-motion prototype trees. Proceedings of the IEEE 12th International Conference on Computer Vision, ICCV 2009.

[44] Viitaniemi V., Laaksonen J., Kollias S.D., Stafylopatis A., Duch W., Oja E. (2006). Techniques for Still Image Scene Classification and Object Detection. Artificial Neural Networks, Proceedings of the ICANN 2006, 16th International Conference, Athens, Greece, 10–14 September 2006.

[45] Papageorgiou C., Oren M., Poggio T.A. A General Framework for Object Detection. Proceedings of the Sixth International Conference on Computer Vision (ICCV-98).

[46] Griffin G., Holub A., Perona P. Caltech-256 Object Category Dataset. https://resolver.caltech.edu/CaltechAUTHORS:CNS-TR-2007-001.

